# Obesity Status and Colorectal Cancer Screening in the United States

**DOI:** 10.1155/2013/920270

**Published:** 2013-04-03

**Authors:** Karima A. Kendall, Euni Lee, Ilene H. Zuckerman, Linda Simoni-Wastila, Marlon Daniel, Pauline M. Green, Beatrice Adderley-Kelly, Anthony K. Wutoh

**Affiliations:** ^1^Science Applications International Corporation, Rockville, MD, USA; ^2^Department of Clinical and Administrative Pharmacy Sciences, Center for Minority Health Services Research, College of Pharmacy, Howard University, Washington, DC 20059, USA; ^3^Department of Pharmaceutical Health Services Research, School of Pharmacy, University of Maryland, Baltimore, MD 21201, USA; ^4^Division of Nursing, College of Nursing and Allied Health Science, Howard University, Washington, DC 20059, USA

## Abstract

*Background*. Findings from previous studies on an association between obesity and colorectal cancer (CRC) screening are inconsistent and very few studies have utilized national level databases in the United States (US). *Methods*. A cross-sectional study was conducted using data from the 2005 Medicare Current Beneficiary Survey to describe CRC screening rate by obesity status. *Results*. Of a 15,769 Medicare beneficiaries sample aged 50 years and older reflecting 39 million Medicare beneficiaries in the United States, 25% were classified as obese, consisting of 22.4% “obese” (30 ≤ body mass index (BMI) < 35) and 3.1% “morbidly obese” (BMI ≥ 35) beneficiaries. Almost 38% of the beneficiaries had a body mass index level equivalent to overweight (25 ≤ BMI < 30). Of the study population, 65.3% reported having CRC screening (fecal occult blood testing or colonoscopy). Medicare beneficiaries classified as “obese” had greater odds of CRC screening compared to “nonobese” beneficiaries after controlling for other covariates (OR_adj_ = 1.25; 95% CI: 1.12–1.39). *Conclusions*. Findings indicate that obesity was not a barrier but rather an assisting factor to CRC screening among Medicare beneficiaries. Future studies are needed to evaluate physicians' ordering of screening tests compared to screening claims among Medicare beneficiaries to better understand patterns of patients' and doctors' adherence to national CRC screening guidelines.

## 1. Introduction

According to the American Cancer Society, colorectal cancer (CRC) is the third most common cancer diagnosed and the third leading cause of cancer-related mortality in both men and women in the United States (US) [1]. While these statistics are alarming, the death rate from CRC has decreased for more than 20 years due to early detection of removable polyps by colorectal screening tests [2]. According to the US Preventive Services Task Force (USPSTF), routine screening may reduce the number of people who die of colorectal cancer [3]. Additionally, treatment for CRC has vastly improved over the last several years, and as a result, there are now more than one million survivors of CRC in the US [4].

The risk of developing colorectal cancer in a lifetime is about 1 in 19 and a number of risk factors associated with CRC have been identified [2]. Established risk factors include increased age, personal history of inflammatory bowel disease, colorectal polyps, and family history of colorectal cancer [5]. In addition, certain behavioral factors such as smoking, heavy alcohol use, and obesity, have shown to be the strongest links to an increased risk of colorectal cancer [2].

Previous studies have reported an association between obesity and a wide range of chronic diseases including cancer. During a 10-year follow-up study by Field et al. [6], the incidence of diseases such as diabetes, hypertension, and heart disease have increased with increasing body mass in both men and women. Moreover, in women, the incidence of colorectal cancer was positively associated with BMI [6]. However, only a few studies have evaluated the association between BMI and screening for colorectal, breast, and cervical cancer [7–11]. BMI status and its associations with the rates of CRC screening were reported, but obesity has been reported as both negative [7–9] and positive predictor [11] for CRC screening. Even fewer studies used national level databases focusing on Medicare beneficiaries in the US. As CRC screenings are covered by the US Medicare program as one of the preventive services, an understanding on factors associated with CRC screening can provide important information to the program for improving access to care and reducing health care costs. 

With the literature of conflicting results that obesity has been reported as both negative and positive predictor for CRC screening, the objectives of this study were (1) to describe CRC screening rates by BMI status with a null hypothesis of equal rates of CRC screening by obesity status and (2) to evaluate if obesity is a predictive factor for CRC screening among Medicare beneficiaries in the US. 

## 2. Methods and Procedures

### 2.1. Data Source and Study Population

Data from the 2005 Medicare Current Beneficiary Survey (MCBS) were used in this study. The MCBS data collection began in 1991 conducted by the Office of Strategic Planning of the Centers for Medicare and Medicaid Services [12]. This MCBS data utilizes survey samples of the Medicare population that utilizes stratified multistage probability sampling procedure with three stages of selection [13, 14]. The first stage involves the selection of primary sampling units (PSUs) consisting of metropolitan statistical areas and groups of rural counties. The PSUs are selected with probabilities proportionate to 1980 population strata defined by Census region, metropolitan status, and selected PSU-level socioeconomic characteristics. Two PSUs were selected per stratum. The second sampling stage consists of the selection of ZIP Code areas within each sampled PSU. At the third and final stage of selection, beneficiaries within the sampled ZIP clusters are stratified by age and subsampled at rates designed to yield self-weighting, or equal probability, samples of beneficiaries within each of seven age groups [14]. This MCBS data including survey samples from the three stages of sampling lead to a sample of 15,769 beneficiaries, representative for 39,337,911 US Medicare beneficiaries. 

The MCBS annually produces two files, the Cost and Use and the Access to Care files. Our study used the Access to Care file which includes information on the beneficiaries' access to health care, their satisfaction with this care, and their usual source of care while the Cost and Use file provides expenditure and source of payment data [12]. The use of MCBS data for this study was requested to the Center for Medicare and Medicaid Services through the Research Data Assistance Center [12]. This file also contains a supplement that includes a survey of Medicare beneficiaries, as well as a survey that measures beneficiaries' sources of information regarding Medicare. These variables include, but are not limited to, history of colorectal cancer, history of other cancer diagnosis, history of screening for colorectal cancer or any other cancer, and the beneficiary's usual source of care providers and care settings. The study population of this study includes all survey samples from 2005 MCBS data consisting of all community-dwelling MCBS respondents aged 50 years and older that can be extrapolated to nationally representative estimates of US Medicare beneficiaries when sampling weights are aggregated.

The dependent variable, Colorectal Cancer Screening (CRC), was created using two variables, COLHTEST (surveyed person was given a home test for fecal blood) and COLSCOPY (if the surveyed person ever had a colonoscopy or sigmoidoscopy) from the MCBS survey data. If the surveyed person experienced either event, they were coded as having a CRC screening.

Body mass index calculation (BMI) for each subject was calculated using self-reported height and weight information available in the dataset using the standard formula of “(weight in lb/(height in inches)^2^).” Based on the definition of BMI classification made by the World Health Organization (WHO) [15], each beneficiary's BMI status was determined (Table 1). For analytical purposes, BMI status was divided into the two categories of “obese” and “nonobese.” This study was approved and conducted in compliance with the requirements of the Howard University Institutional Review Board.

### 2.2. Statistical Analysis

The primary goal for analysis was to investigate the association between CRC screening and BMI, while adjusting for potential confounding variables such as age, gender, race, insurance coverage, and usual source of health care. 

Univariate analyses were conducted to produce descriptive statistics. Bivariate analyses were employed to evaluate unadjusted bivariate associations. Logistic regression analysis was used to model multivariable associations between BMI and colorectal cancer screening, while considering other confounding variables. All analyses in the study used weighted data to yield national level estimates reflecting all US Medicare beneficiaries. The weighted estimates were calculated as means or proportions, with the corresponding 95% confidence intervals (CI) and standard errors (SE). All analyses were done using SAS version 9.1 (SAS Institute Inc., Cary, North Carolina).

## 3. Results

In the 2005 Medicare Current Beneficiary Survey, 15,769 beneficiaries were included as sample (weighted estimates are 39,337,911 beneficiaries) aged 50 years and older, that is, eligible for colorectal cancer screening. The majority of these beneficiaries was identified as Non-Hispanic White (78.8%) and were predominantly female (56.2%) (Table 2). About 37% had at least a high school or vocational degree and more than one half of the beneficiaries were married. Most had private insurance (55.9%) as supplemental insurance and about bout 48.9% of the beneficiaries reported annual income of less than $25,000 (Table 2). About 25% of the beneficiaries were classified as obese (BMI ≥ 30) and almost 38% were reported as “overweight” (25 ≤ BMI < 30) (Table 2). Of the total number of participants, 65.3% reported having one of the two types of CRC screening (Table 2).

Table 3 reports predictive factors for a CRC screening. We found a significant association between obesity status and CRC screening. Medicare beneficiaries classified as “obese” had greater odds of reporting a CRC screening compared to “nonobese” beneficiaries after controlling for confounding variables (OR_adj_ = 1.25; 95% CI: 1.12–1.39). Female beneficiaries had greater odds of receiving a CRC screening compared to men (OR_adj_ = 1.24; 95% CI: 1.15–1.34). Gender as an interaction term was investigated in the logistic regression model but was found to not be statistically significant (*P* = 0.860). Age was associated with CRC screening, with beneficiaries aged 65 and older having greater odds of having a CRC screening than younger ones. After controlling for confounding variables, only the “Other” racial group had lower odds of CRC screening than non-Hispanic White beneficiaries (OR_adj_ = 0.74; 95% CI: 0.62–0.88).

Relative to beneficiaries with private supplemental insurance, those with Medicaid or no supplemental insurance had lower odds of screening as well. Beneficiaries with a usual source of healthcare had odds that were about six times greater for reporting a CRC screening (OR_adj_ = 5.64; 95% CI: 4.69–6.80) (Table 3). 

## 4. Discussion

Findings from our study indicate that “obese” patients have greater odds of being screened for CRC than those who were classified as “nonobese.” A report from the Centers for Disease Control and Prevention indicated that 33.8% of adults in the US are obese [16]. While a number of previous studies reported the association between obesity and cancer screening rates in other cancers like breast [17], cervical [10], prostate cancer [18], and colorectal cancer [11], very few studies used national level survey data. Contradicting findings indicating that obese adults were less likely to be screened for colorectal cancer were also reported [7–9], but previously published studies used various types of data source, methodology, age of the study population, and clinical setting. 

Unique features of our study include (1) the use of national level data representing Medicare beneficiaries and (2) the inclusion of all Medicare beneficiaries aged 50 years and older instead of limiting the study population as beneficiaries who are 65 years and older. The beneficiaries who are between 50 and 65 years of age are eligible for Social Security benefits due to chronic and total disability, having end-stage renal disease or having been diagnosed with Amyotrophic Lateral Sclerosis [19]. Our findings not only provide obesity status as a predictive factor for CRC screening to the body of literature but also report a lower level of access to CRC screening in this unique subpopulation of Medicare beneficiaries with specific clinical conditions of chronic and total disability.

Obesity is one of the well-established risk factors for developing of and dying from CRC [5]. With the increased prevalence in obesity, we believe that potential reasons for the higher CRC screening rates among obese beneficiaries from our study may be related to an increased likelihood for obese patients to receive more frequent surveillance while the obese patients visit their physicians for other chronic diseases. However, obesity can be equally recognized as challenges because outstanding health concerns other than cancer screening may be perceived as more urgent matters. It is also possible that the needs for better managing and accommodating obese patients are acknowledged by care providers. In fact, the importance of improving access to care and sensitivity for obese patients was highlighted in a publication made by the National Task Force on the Prevention and Treatment of Obesity with a set of advice to health care professionals [20]. However, it is beyond the scope of our study in identifying the reasons for higher CRC screening rates for obese patients and we believe more research is needed to better understand the relationship between obesity and cancer screening.

*Limitations of This Study*. There are several limitations to our study, primarily due to the data source being self-reports. Given the way the question of whether or not a beneficiary had ever been screened for colorectal cancer was presented in the MCBS survey, there is no indication as to whether or not the beneficiary had been screened regularly as recommended by national clinical guidelines. An affirmation of having a history of colorectal cancer screening may not correlate to regular screening behavior of the beneficiary. Furthermore, the “history of CRC screening” outcome does not give any information as to *when* the beneficiary may have undergone a screening procedure for CRC. As a result, there may be temporal issues between obesity and other CRC screening related variables and therefore, adherence patterns of CRC screening to national guidelines by Medicare beneficiaries cannot be evaluated in our study.

## 5. Conclusions

Our study described the CRC screening rate by obesity status among Medicare beneficiaries using recently available and large national-level survey data. Unlike findings from previous studies reporting lower prevalence of CRC screening among obese or morbidly obese patients, our study findings are encouraging in that obesity status was not found to be a hindering factor, but rather an assisting factor, for CRC screening among Medicare beneficiaries. In order to gain better insight on adherence of CRC screening to national guidelines, future studies evaluating physicians' ordering of screening tests compared to screening claims among Medicare beneficiaries are needed. 

## Figures and Tables

**Table 1:** World Health Organization body mass index classification.

Body mass index	Classification
0–18.49	Underweight
18.5–24.9	Normal
25–29.9	Overweight
30–34.9	Obese
35–40 and above	Morbidly obese

Source: World Health Organization, 2000 [15].

**Table 2:** Demographic characteristics of Medicare beneficiaries aged 50 years and older in 2005*.

Characteristics	% (SE)
Gender	
Male	43.8 (0.4)
Female	56.2 (0.4)
Age	
Less than 65	15.1 (0.4)
65–74	41.0 (0.4)
75+	44.0 (0.4)
Race	
White	78.8 (1.0)
Black	9.2 (0.8)
Hispanic	5.8 (0.5)
Other	6.2 (0.4)
Education	
College degree or higher	19.5 (0.7)
Some college	14.4 (0.5)
High school/vocational grad	37.0 (0.6)
No high school diploma	29.0 (0.7)
Marital status	
Married	51.5 (0.5)
Divorced	11.8 (0.3)
Widowed	29.4 (0.4)
Never married	7.1 (0.2)
Other	0.2 (0.03)
Supplemental insurance	
Private	55.9 (0.8)
Medicaid	17.3 (0.5)
Medicaid + Private	1.2 (0.1)
None	25.6 (0.7)
Income	
Greater than $50 K	11.0 (0.5)
$25 K to $50 K	40.1 (0.6)
Less than $25 K	48.9 (0.9)
Obesity classification	
Normal or underweight	36.6 (0.5)
Overweight	37.8 (0.5)
Obese	22.4 (0.5)
Morbidly obese	3.1 (0.2)
History of CRC screening	
Yes	65.3 (0.6)
No	34.7 (0.6)

*Using the unweighted data including all samples of 15,769 Medicare beneficiaries from the MCBS data, all estimates are calculated from the weighted analyses. (*N* = 39,337,911 beneficiaries).

**Table 3:** Factors associated with CRC Screening among medicare beneficiaries in 2005.

Characteristics	Adjusted OR (95% CI)
Obesity status	
Nonobese	1.00 (reference)
Obese^c^	1.25 (1.12–1.39)^a^
Gender	
Male	1.00 (reference)
Female	1.24 (1.15–1.34)^b^
Age	
75+	1.00 (reference)
65–74	0.94 (0.86–1.03)
Under 65	0.54 (0.47–0.62)^b^
Race/ethnicity	
Non-Hispanic white	1.00 (reference)
Non-Hispanic black	0.97 (0.83–1.14)
Hispanic	0.97 (0.80–1.17)
Other	0.74 (0.62–0.88)^a^
Education	
College degree or higher	1.00 (reference)
Some college	0.89 (0.75–1.07)
High school/vocational grad	0.67 (0.58–0.78)^b^
No high school diploma	0.52 (0.44–0.61)
Marital status	
Divorced	1.00 (reference)
Married	0.90 (0.74–1.09)
Widowed	0.76 (0.65–0.90)^a^
Never married	0.55 (0.45–0.69)^b^
Other	0.16 (0.04–0.64)^a^
Supplemental insurance	
Private	1.00 (reference)
Medicaid	0.53 (0.45–0.61)^a^
Medicaid + private	0.69 (0.50–0.95)^a^
None	0.79 (0.71–0.88)^a^
Income	
Less than $25 K	1.00 (reference)
$25 K to $50 K	1.22 (1.09–1.36)^a^
Greater than $50 K	1.31 (1.05–1.62)^a^
Usual source of care	
No	1.00 (reference)
Yes	5.64 (4.69–6.80)^b^
History of any type of cancer	
No	1.00 (reference)
Yes	1.19 (1.08–1.32)^a^
History of any type of cancer screening	
No	1.00 (reference)
Yes	2.29 (2.11–2.48)^b^

^a^Significance level *P* < 0.05.

^b^Significance level *P* < 0.0001.

^c^The category includes both obese and morbidly obese beneficiaries.
